# Intracranial mass lesions and skin discoloration in the armpits as unusual clues to Erdheim-Chester disease: a case report

**DOI:** 10.1186/s12883-021-02107-w

**Published:** 2021-02-18

**Authors:** Pedro Gustavo Barros Rodrigues, Isabelle de Sousa Pereira, Valter Barbalho Lima Filho, Daniel Aguiar Dias, Paulo Ribeiro Nóbrega, Pedro Braga-Neto

**Affiliations:** 1 Division of Neurology, Department of Clinical Medicine, Universidade Federal do Ceará, Fortaleza, Brazil; 2 Division of Radiology, Universidade Federal do Ceará, Fortaleza, Brazil; 3 Center of Health Sciences, Universidade Estadual do Ceará, Fortaleza, Brazil

**Keywords:** Erdheim‐Chester disease, Neuroncology, Neuroimmunology, Hystiocitosis, Non-Langerhans-cell, Case report

## Abstract

**Background:**

Erdheim–Chester disease (ECD) is a non-Langerhans histiocytosis that results in multi-organ disease involving the skin, bones, lungs and kidneys. Central nervous system (CNS) involvement occurs in about 50 % of patients, and diabetes insipidus, visual disturbances, and cerebellar ataxia are the most frequent neurological signs. We report a case of Erdheim-Chester disease with central nervous system involvement in the form of enhancing intracranial mass lesions with massive edema.

**Case presentation:**

The patient presented with vertigo, ataxia, encephalopathy and pyramidal signs. Diagnosis was suggested by xanthomatous skin lesions and a biopsy was compatible with Erdheim-Chester disease demonstrating xanthogranulomas CD68 positive (clone KP1) and CD1a and S100 negative. Testing for BRAF mutation was negative, which precluded treatment with Vemurafenib. Treatment with steroids and interferon resulted in improvement of neurological signs and regression of edema on MRI.

**Conclusions:**

The diagnosis of Erdheim-Chester disease should be considered in intracranial mass lesions. Xanthomatous skin lesions are a clue to the diagnosis.

## Background

Erdheim–Chester disease (ECD) is a rare non-Langerhans histiocytosis marked by xanthomatous infiltration of tissues by CD1a/S100 negative, CD68 positive histiocytes [1]. Clinical presentation ranges from asymptomatic bone lesions to severe multisystemic disease [2]. The disorder is characterized by a multi-organ disease, with a preference for the skin, bones, central nervous system, lungs, kidneys and perirenal fat (hairy-kidney sign) [2]. CNS involvement occurs in about 50 % of patients, and diabetes insipidus, visual disturbances, cerebellar ataxia, pyramidal and extra-pyramidal syndromes are the most frequent neurological signs [3]. Lesions in the CNS are an independent factor for poor prognosis and are responsible for one third of deaths in these patients [4]. Magnetic resonance imaging (MRI) findings include retro-orbital masses, involvement of cerebellum dentate nucleus, meningeal lesions of the dura and multiple areas of demyelination of both cerebellum and brainstem, as well as suprasellar lesions and nodular masses of the infundibular stalk [4]. We have found no case reports of large multiple large intracranial mass lesions in ECD.

## Case presentation

**Fig. 1:**
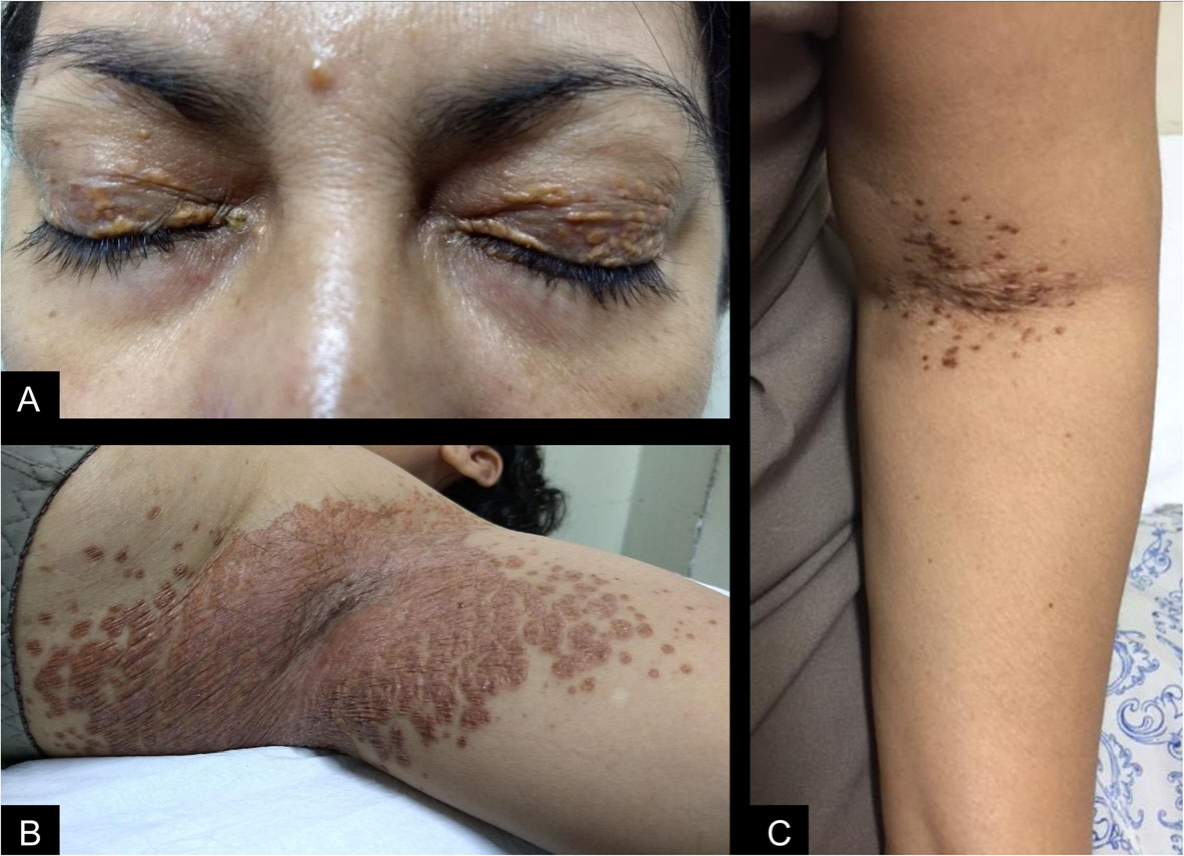
Photograph of skin lesions in a a patient with Erdheim-Chester disease showing yellow plaques in the eyelids suggestive of xanthelasmas (**a**) and confluent, yellow-brown papules in the armpits (**b**) and antecubital fossa (**c**)

**Fig. 2:**
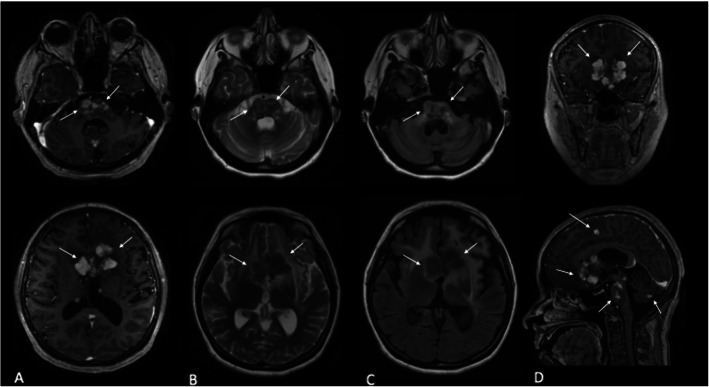
Brain MRI showing bilateral enhancing intraparenchymal mass lesions, most of them located in the pons and anterior basal ganglia (white arrows), surrounded by massive vasogenic edema and predominantly with low signal on T2 weighted images. **a **Axial T1 post contrast at the level of pons and basal ganglia. **b** Axial T2 images. **c** Axial FLAIR images. **d** Coronal (on top) and sagittal T1 (bottom) post contrast images. Note that the lesions sometimes follow perivascular spaces topographies

**Fig. 3:**
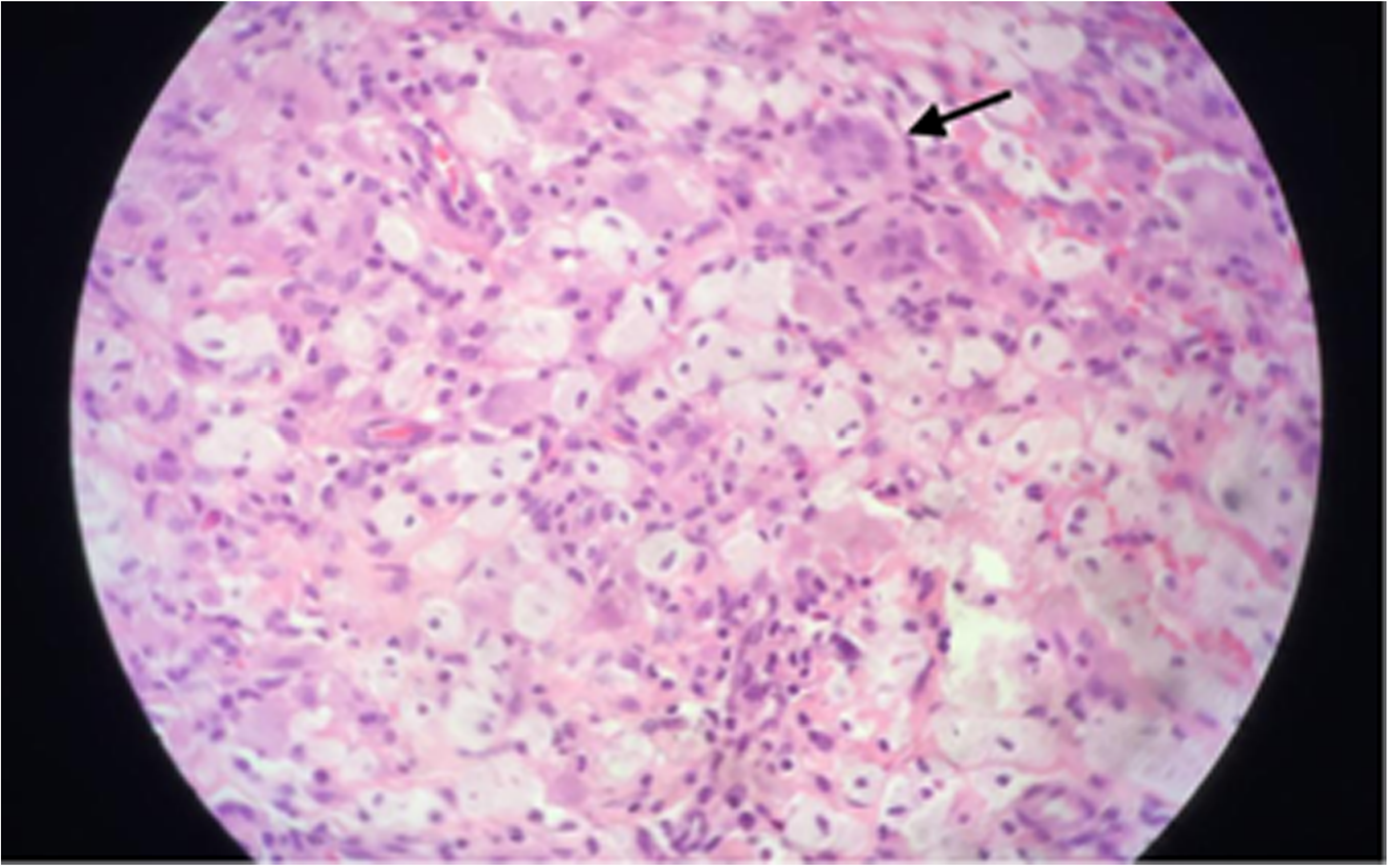
Histopathologic features of a skin biopsy (armpit) in a patient with Erdheim-Chester disease. Skin contains xanthomized and epithelioid histiocytes (bold arrow), some resembling Touton cells, with intermingled eosinophils. No emperipolesis is seen

**Fig. 4:**
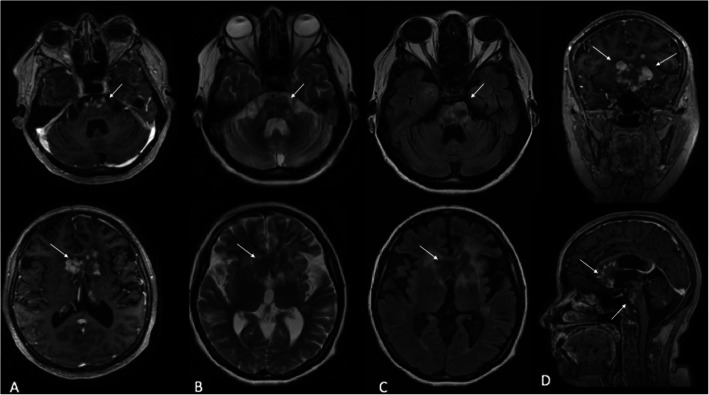
Follow up brain MRI performed 15 months after the first MRI showed in Fig. 1, in corresponding levels and same sequences, showing significant regression of vasogenic edema and partial decrease in lesion size (white arrows) after treatment. **a **Axial T1 post contrast at the level of the pons and basal ganglia. **b** Axial T2 images. **c **Axial FLAIR images. **d** Coronal (on top) and sagittal T1 (bottom) post contrast images

A 42-year-old female patient presented with an 8 month history of constant and progressive vertigo. The symptoms progressed to spatial disorientation and mental confusion, associated with apathy, social isolation and anorexia. Non-pruritic yellow plaques in antecubital fossa, groin, breasts, armpits, eyelids and neck were also reported for the last 5 years (Fig. 1). Physical examination revealed right hemiparesis with bilateral brisk tendon reflexes and bilateral Babinski and Hoffmann signs, left hemi-hypoesthesia, bilateral dysmetria, dysdiadochokinesia and ataxic gait. Magnetic resonance imaging revealed multiple supratentorial, infratentorial and periventricular mass lesions involving the basal ganglia, corpus callosum and the pons, associated with extensive edema (Fig. 2). Her blood cholesterol levels were normal. Two fragments of skin and subcutaneous tissue were obtained, one from affected skin of the armpits and another from skin of the groin. Histopathological exams showed infiltration of eosinophils and foamy epithelioid histiocytes and Touton cells in the armpit lesions (Fig. 3) and collections of foamy macrophages, multinucleated giant cells and predominantly lymphocytic mixed inflammatory infiltrate with numerous eosinophils in the groin lesions. Immunohistochemical examination demonstrated xanthogranulomas (non-Langerhans cell histiocytomas), CD68 positive (clone KP1) and CD1a and S100 negative. Such morphological findings confirmed the diagnostic hypothesis of Erdheim-Chester Disease. Testing for BRAF mutation was negative, which precluded treatment with Vemurafenib. The patient was treated with methylprednisolone 1 g / day, during 5 days, and there was improvement of the cognitive state, but not of the referred vertigo. She was then discharged home with prednisone 60 mg/day and interferon (IFN). After 6 months she had a mild improvement in ataxia, vertigo and confusion. Follow-up MRI at 15 months revealed stabilization of the brain lesions with marked improvement in brain edema and mass effect (Fig. 4). The patient had no further neurological deficits and was able to walk unassisted after two years of follow-up, suggesting stabilization of disease progression.

## Discussion and conclusions

To our knowledge, this is the first case of CNS Erdheim-Chester disease presenting with multiple intraparenchymal mass lesions. The main differential diagnosis of multiple enhancing intracranial masses is CNS lymphoma, but restricted diffusion on DWI is usually seen in lymphomatous mass lesions, which was remarkably absent in our case. The atypical findings on MRI were supported by the characteristic skin lesions in the armpits to suggest the diagnosis and perform a biopsy, which confirmed our clinical suspicion.

Systemic corticosteroids and immunosuppressive agents such as azathioprine, cyclophosphamide and methotrexate have been considered the mainstay of treatment for ECD [5], with varying results. More recently, IFN-based therapy has emerged as a reliable option for ECD patients [6]. Response to IFN is the only major treatment predictor of survival, in particular in patients with CNS involvement [7]. Inhibition of BRAF activation by Vemurafenib in patients that harbor this mutation is a highly promising treatment, with dramatic results in small series [8], but our patient was negative for the said mutation. The use of corticosteroids seems to reduce edema acutely and can be used as combined second-line treatment, once monotherapy is not considered effective [5]. There was improvement in clinical condition and in cerebral edema on MRI with corticosteroids and interferon in the present case. We highlight that the diagnosis of Erdheim-Chester disease should be considered in intracranial mass lesions and that xanthomatous skin lesions are a clue to the diagnosis.

## Data Availability

Not applicable.

## Abbreviations

CNS: Central nervous system
ECD: Erdheim-Chester disease
IFN: Interferon
MRI: Magnetic resonance imaging
